# The experience of Greek-Cypriot individuals living with mental illness: preliminary results of a phenomenological study

**DOI:** 10.1186/s12888-016-1051-y

**Published:** 2016-10-06

**Authors:** Charis P. Kaite, Maria N. Karanikola, Foteini J. D. Vouzavali, Anna Koutroubas, Anastasios Merkouris, Elizabeth D. E. Papathanassoglou

**Affiliations:** 1 Department of Nursing, School of Health Sciences, Cyprus University of Technology, 15, Vragadinou str, 3041 Limassol, Cyprus; 2 Department of Nursing, Vocational High School of Nurse Assistants & Laboratory Instructor, Technological Educational Institute of Athens, Cholargos, Athens, Greece; 3 Children’s Hospital “A & P Kyriakou” Oncology Department, Athens, Greece; 4 Faculty of Nursing, University of Alberta, 5–262 Edmonton Clinic Health Academy (ECHA), 11405-87th Ave., Edmonton, AB T6G 1C9 Canada

**Keywords:** Bipolar disorder, Depression, Mental health nurses, Phenomenology, Psychosis, van Manen

## Abstract

**Background:**

Research evidence shows that healthcare professionals do not fully comprehend the difficulty involved in problems faced by people living with severe mental illness (SMI). As a result, mental health service consumers do not show confidence in the healthcare system and healthcare professionals, a problem related to the phenomenon of adherence to therapy. Moreover, the issue of unmet needs in treating individuals living with SMI is relared to their quality of life in a negative way.

**Methods:**

A qualitative methodological approach based on the methodology of van Manen phenomenology was employed through a purposive sampling of ten people living with SMI. The aim was to explore their perceptions and interpretations regarding: a) their illness, b) their self-image throughout the illness, c) the social implications following their illness, and d) the quality of the therapeutic relationship with mental health nurses. Participants were recruited from a community mental health service in a Greek-Cypriot urban city. Data were collected through personal, semi-structured interviews.

**Results:**

Several main themes were identified through the narratives of all ten participants. Main themes included: a) The meaning of mental illness, b) The different phases of the illness in time, c) The perception of the self during the illness, d) Perceptions about the effectiveness of pharmacotherapy, e) Social and personal consequences for participants following the diagnosis of mental illness, f) Participants’ perceptions regarding mental health professionals and services and g) The therapeutic effect of the research interview on the participants.

**Conclusions:**

The present study provides data for the enhancement of the empathic understanding of healthcare professionals regarding the concerns and particular needs of individuals living with SMI, as well as the formation of targeted psychosocial interventions based on these needs. Overall, the present data illuminate the necessity for the reconstruction of the provided mental healthcare in Cyprus into a more recovery- oriented approach in order to address personal identity and self-determination issues and the way these are related to management of pharmacotherapy. Qualitative studies aiming to further explore issues of self-identity during ill health and its association with adherence to therapy, resilience and self-determination, are also proposed.

## Background

The ultimate principle of care in the realm of mental health is the development of therapeutic relationship between health professionals and people living with mental health problems, including those living with severe mental illness (SMI) [1, 2]. This in turn requires a holistic understanding of perceptions and needs of people living with SMI [3]. SMI refers to a clinical syndrome characterized by significant disturbances in cognition, emotion or behavior, usually associated with notable and chronic distress or disability in work, relationships or other areas of functioning ([4], p.20). However, research shows that comprehension of the problems faced by people living with SMI is difficult for health professionals to achieve, as they hold strong stereotypes regarding mental illness, in line with the wider society. Examples of such stereotypes are that people living with mental illness are unable to communicate their needs, or that their needs are very simple, thus very easy to be understood [5–7]. As a result, people living with SMI do now show confidence in healthcare professionals and provided services [8]. Therefore, an in-depth understanding of the experience of individuals living with mental illness cared by mental health professionals might promote better empathic understanding of the latter [9–16]. This may in turn promote enhancement of the therapeutic relationship, engagement of people living with mental illness in the therapeutic process and finally improvement of clinical outcomes [10, 17–22].

Moreover, during the last decades a particular emphasis has been given on the neurobiological basis of mental health disorders [23, 24]. As a result, pharmacotherapy has been considered as the ultimate treatment method for people living with mental illness [25], along with established guidelines for the implementation of adjunctive psychotherapy and further psychosocial interventions [26]. However, the implementation of medication therapy most of the time does not incorporate the particular concerns and needs of people living with mental illness and their families [27–31]. Therefore, despite the fact that recovery-oriented psychotherapies when applied address issues concerning the quality of life of individuals under psychotropic medication, as well as issues related to their everyday personal and social needs, such issues remain relevant and need to be further explored [28, 29, 32–36].

As for the degree of understanding of the experience of individuals living with mental illness, previous studies focus on specific aspects of this phenomenon [27, 37, 38], thus the number of studies exploring the entire spectrum of the experience of SMI are relatively limited. In particular, a vast amount of literature focuses on involuntary hospitalization during the acute phase of the illness [39, 40] or recovery process [41–44]. Other researchers have explored the coping mechanisms applied by people living with mental illness in order to combat their illness [37, 45, 46]. There have also been studies that focus on the relationship between individuals living with mental illness and health professionals [47–51] and others that focus on the attitudes towards pharmacotherapy [36, 52, 53]. The current study aims to explore the way people living with SMI interpret their everyday life through the spectrum of their illness in Cyprus.

Scientific literature suggests that the way an experience is interpreted by individuals with SMI with regard to ill health, is formed by the particular context in which each person lives [54–58]. Although prior studies address and describe the entire living experience of SMI, these arise from different cultures and health systems. It is therefore important to study whether or not these experiences differentiate within the cultural context of Cyprus, especially since there has been no prior relevant data [59, 60].

The context of mental health services in Cyprus includes both community and hospital services, which are provided in all five geographical areas of Cyprus. These services focus on the prevention and early diagnosis of mental illness, as well as early intervention in order to prevent the chronicity of mental illness [61]. Hospital services include a) the psychiatric clinics of two major general adult hospitals (Nicosia and Limassol hospitals) and b) Athalassa psychiatric hospital for adults in Nicosia. The main community units are: a) community mental health centers, including out-patient clinics integrated in the urban health centers of the general hospitals, b) child and adolescent mental health services, c) units for psycho-social rehabilitation (day centers and vocational rehabilitation units), and d) prevention and treatment units for drug abuse [61]. In recent years several changes have been made to mental health services in Cyprus which highlight the need for studies exploring issues related to the quality and safety of care provided by these services. Specifically, the majority of mental health services have been transferred from institutional to community settings. As a result, the number of patients in the Athalassa psychiatric state hospital, which is Cyprus’ major mental health institution, has been reduced dramatically, from 584 patients in 1990 to 105 in 2011, leading to an increase in community mental health service consumers [61]. International data suggest that a marked change in the form of mental health services may result in increased workload for the community mental health professionals, as well as greater complexity of the needs of the consumers of community mental health services [19, 62]. The aforementioned conditions, as well as lack of nursing specialist roles to address the intricate needs of people living with mental illness [63], within the context of advanced clinical practice as an imperative part the therapeutic inter-professional approach, may have influenced the quality and safety of the delivered mental healthcare and its effect on the experience of individuals who suffer from SMI. Advanced nursing practice regards a highly developed level of provided nursing care in terms of clinical experience and knowledge, clinical assessment and therapeutic approaches [64]. International literature confirms that advanced clinical nursing practice in the community is followed by lower rate of relapse and morbidity and higher standards of quality of life [65, 66]. To our knowledge, there is no evidence to which extent recovery-oriented approaches have been incorporated to the recent transformation of mental health services in Cyprus [61].

Under this scope, it would be interesting on the one hand to explore for the first time in Cyprus, not only after deinstitutionalization but also before it [67–74], how people living with SMI internalize their illness and the services provided in order to highlight their main concerns, and on the other to provide information and particular directions for future studies in relation to these issues.

Within the Greek and Cypriot context there have been studies addressing the concept of care in general [60], as well as attitudes and beliefs of healthcare professionals or those of the general population [75]. Additionally, there is a call in Cyprus from the Ministry of Health regarding the necessity to educate the public about the problems and needs of people living with mental illness. The ultimate aim of this call is to confront and combat prejudice and social stigma of mental illness by increasing the mental health literacy of the public. The collection of data about these issues is included in the aims of the current study [61]. Moreover, taking under consideration the goals of the Ministry of Health of the Republic of Cyprus regarding mental health services, the current study is expected to contribute to the promotion of the quality and safety of the services provided, since it focuses on the individualized needs of the consumers of mental health services.

### Aim

The aim of the current study is to explore the perceptions and meanings of Greek-Cypriot individuals living with mental illness in relation to: a) their illness, b) their self-image throughout the illness, c) the social implications following their illness and d) the quality of the therapeutic relationship with mental health nurses.

According to the principles of phenomenology, as a qualitative research method, the aim of a study should be to find answers to questions such as “What is it like, to live with mental illness?” or “What is the meaning of an experience, within the mental illness?” Such research is designed to explore the phenomenon of interest without preconceived notions or hypotheses so as to reveal unknown perspectives of it [76]. Overall, research questions based on phenomenology allow participants to describe their experiences as deeply as they wish to. They also provide space for an in depth analysis of the narrative of each participant in order for concealed parts of an experience to be revealed [77].

## Method

### Study design

A qualitative phenomenological approach based on the methodology of van Manen was employed. According to van Manen [78], phenomenology is the study of the world as experienced in everyday life situations and relations (lifeworld), particularly as it is experienced by those who live it, not as it is perceived by the researcher. With regard to health and illness, the focus of phenomenology is the description and revelation of the phenomena of caring and healing, as well as the living experience of individuals who are part of these procedures [79–84]. The method of van Manen is both descriptive and interpretative (hermeneutic) [85]. Thus, the aim of studies applying the phenomenological approach is to explore the subjective interpretation of the living experience of individuals and unveil their meanings and perceptions regarding the experienced phenomenon through their lifeworld stories [86].

Specifically, the van Manen method entails the description of how one orients oneself into a phenomenon, for example how one describes the different phases or stages of an illness. Also, focuses on the interpretation of the living experience with this phenomenon in its essences [85], for example how one perceives oneself within the different stages of an illness. The term “essence” refers to the central meanings of a phenomenon which distinguish it from another [87]. The application of the methodology of van Manen [87] includes several distinct phases. The first one refers to all actions the researcher takes in order to select a particular phenomenon under study and form the proper research questions relative to the aims intended to be explored. The second phase is known as the “existential investigation” and concerns the study of any available source of data that may help the researcher to expand his/her awareness regarding the subject under study. The sources that may be used include: a) literature, b) poetry, c) songs, d) art and e) movies. The next phase regards the conduct of direct personal interviews with those who have an experience related to the phenomenon under study [88]. The data collected in that phase are analyzed during the last stage of this methodology, which is known as “Hermeneutic Phenomenological Reflection”. Overall, the methodology of van Manen is widely used in nursing literature [85, 89–93].

### Sample

Participants were recruited from a community mental health service, in one of the four urban Greek-Cypriot cities, during the period between April of 2012 and August of 2013. The facilities provided by this particular service to individuals living with mental illness were: a) music therapy, b) drama therapy, c) computer lessons, d) drawing, e) exercise, f) arts and crafts, g) counselling and h) occupational therapy. Moreover, psychoanalysis was provided for individual level analysis, psychodrama was used for either individualized or group level and lastly Cognitive Behavioral Therapy was applied to both individualized and group level. These approaches depended on the individualized needs of consumers of mental health services, and were mainly used to confront: a) trauma, b) diagnosis, c) relapse episodes and d) consequences of psychopathology.

During the period of data collection, the community mental health service from which data were collected, included 40 individuals, the majority of whom had a diagnosis of psychosis or mood disorder and were unemployed. However, the average daily number of people visiting the service during that period was about 20. This was due to the fact that some of the users of the service were in relapse experiencing severe symptoms of pathology. They were either hospitalized or incapable of participating in the activities of the study setting. Eventually, these people were unable to visit the service, even if they wished to and did not participate in the study. Overall, participants were chosen through purposive sampling according to the criterion of theoretical saturation of emergent themes, since data collection and analysis were taking place simultaneously [94]. The enrollment of new participants was finalized when no more novel themes were arising according to data analysis. Moreover, the following inclusion criteria were set as follows: a) a diagnosis of psychosis, mainly schizophrenia, and/or mood disorder, mostly bipolar disorder, according to DSM- IV-TR [95] or ICD-10 [96], b) voluntary use of the community mental health services, c) age of over 18 years, d) fluent knowledge of the Greek language, e) ability to reflect on the living experience and describe it to the researchers, as it was drawn from the narrative content, as well as score 20 or more in the MMSE [97], and f) ability to describe personal experience in depth followed by relevant examples. The exclusion criteria were: a) being in relapse, thus hospitalized, b) showing inability to reflect on personal experience due to severe cognitive and sensory perception disturbances, c) severe impairment of cognitive functioning, d) diagnosis of Axis II of DSM-IV-TR [95], e) poverty of speech, f) lack of insight, g) insufficient understanding of the content and goals of the study. Finally, 10 people out of the 20 visiting the clinical setting of the present study responded to all the aforementioned inclusion criteria and were enrolled in the study.

### Data collection

Data collection was achieved through phenomenological semi-structured interviews based on a guide developed according to relevant literature [8, 67, 98–101]. The guide included open-ended questions, and had been given to a panel of experts in order to evaluate the appropriateness of each question prior to the study. The panel of experts consisted of four distinguished academics in the fields of qualitative research, mental health and research methodology, two being Associate Professors one Assistant Professor, and one working in University. They all have extensive published research work on the topic of the present study. The comments of the panel of experts included the elimination of a number of questions, as well as the simplification of the language used. However, each participant had a significant contribution to the final form of each interview, according to the rules of phenomenology [87]. The questions included in the guide were:

1. “Please describe to me how it is to live with mental illness”.
2. What does it mean to you to live with mental illness?
3. “How do you perceive yourself in the context of living with mental illness/How does living with mental illness affect your thoughts/What do you think about yourself?”
4. “What is your everyday life like living under the scope of mental illness?”
5. “What are your experiences from your contact with nurses and/or how would you describe your relationship with nurses?/What kind of feelings do you experience from your contact with nurses?”

Moreover, participants were encouraged to refer to examples of their experiences to enable the in depth understanding of their meaning. At the end of each interview, demographic data were collected (e.g. sex and age), not only to highlight the profile of the participants, but also to draw attention to possible variations in the emerging themes according to special features of the participants. Both verbal and non-verbal messages were transcribed. All interviews were carried out by the primary investigator. The focus was on the living experience of SMI and self-image. Specifically, 2 interviews were conducted with each participant ranging from 1 to 3 hours in duration. When data analysis was completed a third interview, aiming to discuss the results of the study with each participant, took place. Thus, during the second and third interview the emerging themes were validated and enriched.

### Data analysis

Data analysis was performed by four independent researchers: a) the main researcher, a Doctor in the field of qualitative studies (CK), b) the second researcher, an Assistant Professor in Mental Health Nursing, having a long experience and subsequent number of publications in phenomenological analysis (MNK), c) a Doctor in nursing with experience in phenomenological analysis (FV) and d) an MSc psychologist with experience in phenomenological analysis (AK).

Phenomenological data analysis, according to van Manen [87], aims to uncover the structural or thematic aspects of the experience of a phenomenon, and grasp the essential meanings of a phenomenon under study. The essential meanings are what uniquely describe the phenomenon under study, including the aspects or qualities which make a phenomenon what it is and without which the phenomenon could not be what it is [87].

During the first step of the analysis, researchers read the text of the transcribed narratives several times. They then underlined sentences or words that seemed to reflect meanings, perceptions or feelings which they considered particularly essential for analyzing the phenomenon under study, i.e. the experience of mental illness. In other words, the researchers tried to find quotes from each narration that seemed to be meaningful, thematic and capable to unveil perceptions and feelings experienced in the context of mental illness. The second step was to identify whether those quotes were associated with one another, in order for the researchers to identify groups of quotes under particular thematic sections. This process led to the development of two main thematic sections, one regarding perceptions and feelings about the illness and its therapy, and the other about perceptions and feelings about the self. Moreover, a list of themes derived from these two groups of quotes. These were the main themes, whilst some of them included further subthemes. Next, each researcher wrote down the main themes or subthemes he or she had identified and validated them with a representative quote [87]. The finalization and naming of the main themes were obtained from the consensus of all four researchers who participated in data analysis.

The final step of the phenomenological analysis was to identify the essential themes that uniquely describe the phenomenon under study and are nonetheless significant for the living experience. Thus, essential themes reflect on the aspects or qualities that make the phenomenon what it is and without which the phenomenon could not be what it is [87]. The present study concerns the preliminary results of the exploration of the living experience of SMI, since the analysis of the essential themes is still ongoing. As a result, the essential themes will not be presented herein. Instead, the main themes and subthemes of the analysis are presented in the section of the Results.

Finally, the researchers presented the participants with the themes they had considered to be important in relation to the phenomenon under study. They then asked the participants the following question “Is this what the experience is really like?” in order for the participants to confirm the interpretations of the researchers. Through this procedure the main themes and subthemes were further validated and enriched, as mentioned earlier [87].

### Human subject issues

The study protocol was approved by the Cyprus National Bioethics Committee (File number: 2011.01.39).

Approval from the Research Committee of the Ministry of Health of Cyprus was obtained in order to gain access to all outpatient mental health services over the island of Cyprus (File number: 5.34.01.7.3E), as well as from the Office of the Commissioner of Personal Data Protection (File number: 3.28.23) [98]. Participants were informed of the purpose and procedure of the study prior to their consensus to participate (article 11(1), Law 138 (1)/2001) [102], and all the participants finally included in the study signed an informed consent form.

With regard to ethical issues in data collection, all actions were taken into consideration in order to preserve anonymity and confidentiality. In particular, all the participants, who met the inclusion criteria, read the informative consent form and were given the opportunity to ask the primary investigator further questions about the research before signing it. The main researcher had the responsibility of collecting the informed consent forms.

Moreover, interviews were coded. Prior to the beginning of each interview participants were asked to choose a pseudonym in order to participate in the study, establishing their anonymity. Thus, the identity of the participants was not revealed anywhere during the study or in any other material related to the study.

Interviews were tape-recorded, unless a participant had not consented to. They were carried out at a place and time chosen by each participant, in a comfortable environment, where there was no chance of any intrusion by a third party, thus establishing privacy and preserving confidentiality. All tape-recorded interviews were safely kept in a locked drawer at the office of the assistant professor MNK.

## Results

Ten individuals agreed to participate in the study, two females and eight males. The age of the participants ranged between 26 and 65 years, the majority of whom being unmarried. One was divorced and three of them were married with children. Four of the participants had a primary school education and the rest had a secondary school education. Most of the participants were not employed, whilst one was retired. Only one participant ran his own business. With regard to diagnosis, the majority were diagnosed with psychosis, mainly schizophrenia and three of them with Bipolar Disorder.

In connection with the way the presentation of the results was organized, main themes and subsequent subthemes were reported following each study objective. In particular, two main themes were identified with regard to the first objective, which considered the perceptions of the participants about mental illness. The first theme was “The meaning of mental illness” with subthemes: a) the unbearable mental illness and b) the pseudonym)”, while the second theme was “The different phases of the illness in time”. With regard to the second objective of the study namely the participants’ self-image throughout the illness, two main themes were also identified: a) “The perception of the self during the illness” and b) “The bargaining about the effectiveness of pharmacotherapy during ill health”. Regarding the third objective about the social implications following their illness, the main theme was “The social consequences following the diagnosis of mental illness”, followed by two subthemes: a) The demeaning of self-value in people living with mental illness by society and b) Adjustment of personal and social needs. With respect to the fourth objective of the study concerning the quality of the therapeutic relationship with mental health nurses, one main theme emerged, namely “The perceptions of the participants regarding mental health professionals and services” with two subthemes: a) The Community Mental Health Centers as a means for happiness and safety, and b) The relationship with the PHMNs as a dynamic process. An additional theme identified was “The therapeutic effect of the research interview on the participants”, though this was beyond the scope of the current study.

### Main themes and subthemes

#### The meaning of mental illness

##### The unbearable mental illness

As far as the meaning of mental illness is concerned, participants contributed long, vivid and emotional descriptions of their experience of mental illness, which revolved mainly around suffering, trauma and pain, bringing into light the unbearable character of mental disorder. Overall, it seemed that the way participants lived through their condition was a traumatic experience, as it was drawn from both their narratives and non-verbal cues. Specifically, they compared their experience with the martyrdom of Jesus Christ, or the martyrdom of people who have lost their legs and hands.

*Lion: “My life has been* ***traumatized*** *…”*

*Kostas Theofanous: “It is better to get* ***amputated*** *than to be mentally ill”.*

*Christos Anesti: “Ongoing* ***suffering*** *[…]”*

##### The Pseudonym

Although it is uncommon to interpret the pseudonyms participants choose for the purpose of a research study, the researchers of the current study could not help noticing both the captivating choice of pseudonyms, as well as the powerful illustrations participants provided for them. Thus, they were compelled to interpret the hidden meaning or connection these pseudonyms might have had with the living experience of the participants through mental illness.

Participants seemed to select a pseudonym, linked either to their living experience of illness, or for most of them it appeared to be related to their expectations regarding the clinical outcome of the illness. Thus, the pseudonym seemed, partially to represent the participants’ indirect but unique way to express their interpretation of the illness.

*Christos Anesti: “Jesus Christ was resurrected, and it crossed my mind to use this pseudonym, coming from Christ, that is to say Christos Anesti (meaning resurrected). […] With the help of God and pharmacotherapy […] these thoughts of the tormented person, of the tormented Christos will go away.* ***And when all these (symptoms) will have gone away*** *, there will be* ***resurrection for me*** *. Christ was crucified. He suffered a lot on the Cross, and He was humiliated. He lived the experience of humiliation. And after His martyrdom,* ***He was resurrected*** *. That is to say He went to Heaven […] Now, He is a light. […] I will feel the same way, about the same. I will be resurrected.”*

Selecting Christos Anesti as pseudonym, this participant seemed to equate the course of his ill health, described by him as “ongoing suffering” (see his previous quote) with the course of the testimony of Christ. Indeed, the participant described himself as “tormented” when he was asked “how do you feel having this illness?” In addition to the way the participant interpreted his illness, his choice of this particular pseudonym seemed to reflect his anticipation of the clinical outcome of his illness. The term “resurrection” and “tormented” quoted above are representative of the participant’s inner suffering.

The relationship between the pseudonym chosen by the participants and expectations and meaning of mental illness might also be highlighted in the pseudonym “Lion”. Based on the participant’s narrative, his expectation about himself through the illness was to be strong, like the king of the Jungle. The term “Jungle” probably implies the difficulties and possible “traps” and dangers that the diagnosis of the illness involved.

*Lion: “Lion I think is the* ***King of the Jungle*** *, and I think that I can be classified as a Lion in a way […] and I want* ***to be very strong, (during illness) and I will do everything about this.*** *”*

#### The different phases of the illness in time

Participants described concretely the different phases of the illness in time making a clear distinction between a) the past, reflecting on the condition they were in before the beginning of treatment, b) the present, reflecting on how they felt their state during the treatment phase, and c) the future with their expectations of themselves and the outcome of the illness.

During the healing phase, all participants reported that it was through medication, their own initiative and spirituality that a change would be achieved. Regarding recovery phase, which concerns the future, it appeared that the participants expected their integration into the community to be long and gradual. They also hoped for normality.

*Christos Anesti: “[…]* ***since I started taking that medication*** *, those (symptoms)* ***have gone away*** *, that feeling of my brain being muddy… my thoughts being muddy, and my soul […] so sad.* ***Now I feel differently*** *. I do not feel that way. I believe that gradually, and with the help of God, and if I find a nice girl, I* ***will become well*** *. I will become a normal, a perfect person.”*

#### The perception of the self during the illness

The way in which participants perceived themselves during the illness was revealed through descriptions such as **“tormented”, or person of “bad character”,** mainly reflecting the way they were feeling about themselves when the symptoms of the illness were in relapse.

*Christos Anesti: “[…] it will be like a resurrection […] the thoughts of the* ***tormented*** *person will go away […]”.*

*Vivi: “[…] I attend a rehabilitation program in order to improve my existence, my* ***bad character*** *because of which I have been* ***tormented*** *for so long”.*

Overall, the descriptions revealed that participants were perceiving their “self” as being devalued and dehumanized due to the illness and subsequent treatment, leading to an altered self-identity and demeaning self-appraisal.

*Vivi: “I have entered the Psychiatry world my darling and… Ahhh! Since then, myself had never been at that level (of existence), as that of a young woman, or a spouse. (My self) was at the level of* ***taking pills and antipsychotics, myself has felt devalued*** *and has gone through so much to reach the point to make peace with myself […]”.*

#### The bargaining about the effectiveness of pharmacotherapy during ill health

Throughout the interviews it was evident that all participants were in an ongoing strive to accept the benefits of medication treatment. The degree of acceptance of the benefits of pharmacotherapy seemed to fluctuate through time according to the different phases of the illness, the perception of self, as well as the level of self-esteem. At times, the participants were overwhelmed by both the side effects of medication and the stigma related to psycho-pharmacotherapy, whilst they often described pharmacotherapy as the ultimate prerequisite for integration to society and normality.

*Lion: “If I don’t take my pills, I feel bad. […] Now I feel very good. […] I believe it is the pills. That I must take them as prescribed. […]* ***They help me, but I can't accept the fact that I take pills*** *[…] I can’t accept it. Even the injection has an effect on me. […] I take the pills and my physician tells me ‘Use the injection and stop the pills’. This is not right. I should take fewer pills”,*

The narratives also revealed that adherence to medication was associated with the construction of the “mental patient” identity and the loss of the so far structured personal and social identity. Overall, participants described that the illness was formed into an experience, mainly a suffering one, when they started receiving psychotropic medication. The main reason for this notion seemed to be social stigma, as well as inability to perform ordinary, social roles.

*Vivi: “* ***I didn’t want to*** *. (to start medication) […] to be part of the sick ones […] I wanted to be a woman, a lover, a young mom […]* ***due to these injections, I am attending a rehabilitation program.*** *[…]. I am improved. From being a log to a normal human being. To a person who has found his legs, his hands, himself. I had lost all these. I had lost my mind. I was in panic, I had lost myself. Ohhh! I lost Vivi who was helping herself, who was feeling nice”.*

#### The social consequences following the diagnosis of mental illness

##### The demeaning of self-value of people living with mental illness by society

Participants described several consequences due to their illness. The main one involved social stigma and subsequent labeling. Specifically, participants described particular factors contributing to the social stigma of mental illness. These included the “extra-ordinary” character of symptomatology of mental disorder, prescription of psychotropic medication alongside with its side effects, and mainly the custodial psychiatric hospitalization in Athalassa psychiatric hospital, the place where involuntary admissions take place. Even the benefit from social welfare was described as a factor associated with the labeling of mentally ill people.

*AEL: “when someone is hospitalized, the community labels him as “crazy”, “lunatic”. Oh!* ***He has been to Athalassa, he is completely lunatic, he is scum, he is a pothead*** *. Similarly, if you are mentally ill, all of them (the society)[…] they stigmatized you.* ***The Society. And even the State, sometimes, with the social welfare benefit, (meaning) that you are unable to work*** *”.*

Experiences of self-value being diminished by family members were also described by the participants.

*Vivi: “My husband […] he wants* ***to demean me*** *, as being a person with mental illness […] He doesn’t even want me to go to his office. […] I am the sick one,* ***I am the demeaned*** *of the house, I* ***am the one who takes antipsychotic pills, the one who is being given injections*** *.”*

##### Adjustment of personal and social needs

Within the framework of the consequences of mental illness, most of the participants described an ongoing struggle to adjust their personal and social needs to the particular limitations set by their illness. Basic needs, such as a normal sex life, companionship and reciprocity or belonging to a group of people were “absurd” for people with mental illness.

*Kostas: “I try to make* ***my utmost to be reasonable*** *. Not to ask for absurd things, such as women for sex.”*

Moreover, while the need of vocational rehabilitation seemed to be related to the need of belonging to a group or being an equal member of society, at the same time indirectly conveyed the sense of being excluded from society.

*Vivi: “[…] I wanted a title for myself too, even that of a beautician or a hairdresser, to have a title of any kind, a job,* ***to be part of a society.*** *[…] And I would also go even to a supermarket to work.”*

#### The perceptions of the participants regarding mental health professionals and services

##### The Community Mental Health Centers as a means to happiness and safety

The participants used constructive and encouraging descriptions about the quality of services provided in the community. The therapeutic character of the community mental health services was described in terms of socialization, creativity and rehabilitation.

*Kostas: “I come here (Day Care Center) to avoid the bad experiences coming from the outside world, like laughing at you […] and to avoid the psychiatric unit. […] Rather than being in the Psychiatric hospital (Athalassa) […] I attend the rehabilitation group. And they (health professionals) had to start from zero […] we had become like babies, completely babies and we have become persons almost like persons. Yes,* ***I have a good time here*** *[…] and* ***I keep company.*** *”*

In contrast, the descriptions of Athalassa psychiatric hospital were as tragic as their concept of the illness. However, it must be noted that acute psychiatric care, including involuntary admissions, takes place solely in Athalassa hospital.

*AEL: “The climate that prevailed in Athalassa? [..] Little food. Many pills, many injections. I was feeling awful. A ruin, drooling, you were in dirty clothes. Bath? Their bathrooms were ancient. You felt disgusted to have a shower. Yes,* ***even now the situation is very tragic*** *.”*

##### The relationship with mental health nurses as a dynamic process

Most of the participants described a developmental process regarding their relationship with mental health nurses, which included different phases and emotions analogous to their illness, such as anger and conflict, withdrawal and isolation and finally support and coziness.

*Mother: “I was yelling at them (nurses) because of my illness […] and I was arguing with them […] and they told me to stay home for a while, […] until I was no longer angry […] and I didn’t go there for two months. Today, nurses […] are close to me, they ask me how I am doing and I tell them everything […]”*

#### The therapeutic effect of the research interview on the participants

The participants expressed their need to communicate their experiences, concerns and feelings about being mentally ill, a phenomenon evident in their narratives. The given emphasis on the therapeutic nature of the procedure of the research interview by the participants, although significant, was beyond the intention of the study. The need to be heard was evident in the participant’s narratives.

*Kostas Theofanous: “I would like to thank you too, because you accepted me. Yes, because I described my feelings to you and I remembered the past… And I expelled some residues off me, which were useless. When I talk, I calm down. I want neither to have a cigarette, nor anything”.*

Interestingly, some of the participants expressed their desire for the results of this study to be distributed to the members of their therapeutic team, in order to enhance understanding and empathy of mental health professionals regarding the experiences of the participants through their illness.

*Christos Anesti: “[…] (psychiatrists) and now that I have talked to you I feel recovered. I want to recommend something. Give them (psychiatrists) the findings of this study”.*

## Discussion

Since the aim of the present study was to qualitatively explore the experience of Greek- Cypriot individuals living with mental illness, it should be noted that this is the first study, to our knowledge, which investigates this issue within the cultural context of Cyprus, both prior to and after deinstitutionalization. In particular, this study took place under the recent psychiatric reform in Cyprus, which resulted in a change in the type, number and staffing of mental health services. The main findings of the study regard: a) the meaning of mental illness as an unbearable, suffering disease, b) the perception of the self during the illness and how this is associated with adherence to pharmacotherapy and subsequent clinical outcome of the disease, c) the perception of different phases of the illness in time, d) perceptions about the effectiveness of pharmacotherapy, e) social and personal consequences for the participants following the diagnosis of mental illness and related social stigma, f) the different phases of the therapeutic relationship between patients and mental health nurses in time, and g) the quality of provided mental health services as perceived by people living with mental illness.

### The meaning of mental illness, its therapy and perception of the self

With regard to the meaning of mental illness, a painful, suffering and multifaceted experience was described herein by the participants, congruent to previous studies [54, 103–107]. Participants clearly described two distinguished sources of suffering; firstly the distressing experiences stemming from the pathophysiology of the disease and side effects of pharmacotherapy and secondly social stigma associated with mental illness and psychotropic medication. The unbearable and multisided pain following the onset of mental disorder symptoms has also been previously described in international literature as a catastrophic experience which interrupts one’s life [108–112]. However, the concrete differentiation among the main sources of suffering reported herein has not been formerly discussed in depth in the literature, and may further be useful in education of healthcare professionals regarding the recovery model and its implementation to mental healthcare [113], as well as in the way recovery-oriented psychotherapeutic interventions may be organized. For example, a key target of such approaches may be the alleviation of distress following SMI diagnosis and possible consequences of relevant trauma, as well as the empowerment of people living with SMI through the periods of suffering [114]. An additional group of interventions may encompass pharmacotherapy-related psychosocial techniques and the effective management of its adverse events under the scope of patients’ wishes and needs [115, 116].

Most importantly, these findings reveal the limited implementation of recovery-oriented approaches in the healthcare system in Cyprus, since the participants appeared to suffer strongly due to their illness, while their re-engagement into everyday routine seemed to be only partially achieved [117]. In contrast, the key principles of recovery-oriented healthcare systems determine that individuals living with SMI are viewed as facing chronic, rather than acute, problems which necessitate long-term support with emphasis on recovery management rather than disease management [117]. The focus is on the enhancement of a positive self-image and self-determination, remission of symptoms and systematic empowerment for a normal and qualitative way of living. Overall, recovery-oriented approaches focus on inspiring hope in people living with SMI and their families, person-centered care and finally the needs and perspectives of mental health services consumers [118].

The lack of recovery-oriented system in mental healthcare in Cyprus may also explain the way the participants perceived themselves during the illness [119]. The descriptions they chose to portray themselves were mainly negative ones, for example “bad person” or “tormented person”. Such descriptions seem to reflect the participants’ diminished self-esteem, stemming probably from inadequate implementation of interventions which aim to enhance their resilience and positive personal identity traits. [114] One may argue, by contrast, that negative self-image may also rise from the common universal stereotypes often attributed to mentally ill people, such as “dangerous” or to their disease as “untreatable” or “bizarre” [68]. Research shows that most of the times the public describes the majority of chronic illnesses, such as cancer or heart failure, as “bad situations”, giving emphasis on the condition related to the disease [105], as opposed to mental disorders, where emphasis is given on the person suffering from it. This discrimination gives ground to labeling and social stigma, consequently affecting the self-image of individuals living with mental illness [67, 105].

In line with the above, we also suggest that the fact that the majority of the participants chose a common Greek-Cypriot name or surname as a pseudonym may to a degree reflect on the participants’ need not to aspire to the identity of the mentally ill person. Hence, their illness would be perceived as a common one, unable to influence their personal identity and esteem in a negative way. Prior literature illustrates the need of mentally ill people to preserve their self-identity prior to illness, and consequently their uniqueness and individuality beyond the psychiatric diagnosis [24, 116]. Moreover, data shows that loss of self-identity is strongly connected to the onset of mental illness, and intensified by social and self stigma [120, 121]. The study by Wisdom et al. [111] exploring identity-related themes in individuals living with SMI revealed that these people experience loss of their self due to the onset of mental illness, having the sense that they are either dead or that they have lost a particular part of their identity, mostly connected with their social roles e.g. being a parent. Additionally, Wisdom et al. [111] reported that people living with mental illness often describe their self as a stranger when they refer to their existence during the illness, while they also mention a perception of duality of self in terms of the sick and the healthy self. Based on the results of the present study and related literature, we propose that interventions targeted at helping people maintain positive self-identity and self-appraisal through the different phases of mental disease is of utmost importance.

The way mentally ill people internalize their self during the illness and the degree to which this perception influences their self-esteem and self- image appears to be a really important issue, since it seems to be related to adherence to therapy, both psychosocial and pharmacological and subsequently to clinical outcomes [103, 122]. Based on such data, the findings of the present study highlight the need for recovery-oriented approaches focused on the enforcement of positive personality values, such as positive self-appraisal, motivation and hope in order for the Cypriot consumers of mental health services to gain the desired quality of life [122]. In more detail, the participants of the current study described that the symptoms of their disease had affected their existence negatively. They also stated that it was mainly through pharmacotherapy that their symptoms had gone away, and hence were able to attend rehabilitation programs and further reintergrate into society, gaining a perception of positive self-value. Overall, one may argue that participants’ need to preserve a positive self-perception was both the main buffer against the negative effects of pharmacotherapy and the ultimate means of adhering to pharmacotherapy, as they did not mention any psychotherapeutic approaches provided to them towards this goal. In contrast, along with the effectiveness of pharmacotherapy, the participants underlined personal initiative and effort against the limitations set by the illness as a prerequisite for recovery.

At the same time, participants described that engagement into pharmacotherapy was also associated with demeaning self-appraisal, mainly due to social stigma and subsequent self- stigma. As a result, participants described an ongoing struggle to accept the effectiveness of medication, however, without, mentioning any kind of support through this process. This ongoing struggle was supported by the positive effects of pharmacotherapy on self-perception and functionality, as well as stigmatization and social withdrawal. Participants characterized stigmatization as an important consequence of being prescribed psychotropic medicines, which caused their transition into the group of “sick people”. It seemed that pharmacotherapy in some way evoked the formation of the identity of “mental health patient” for the participants, a fundamental reason for them to drop out of therapy. Based on the above, one may underscore the lack of interventions to effectively address issues associated with sigma within the Cypriot cultural context, both social and healthcare related, in order for people living with SMI to achieve quality of life [123].

Nonetheless, the necessity of medication to control symptoms, improve cognitive functioning, prevent relapse and promote recovery has been stressed in international literature [73, 120, 121, 124–127]. However, this requires effective management of pharmacotherapy and relevant adverse effects, and, mainly, participation of mental health services consumers in the development of the therapeutic plan [99]. Interestingly, Piatt et al. [36] underlined medication as a means of transformation of self and further as a major component of recovery, although they did not address the way medication may empower self-view and self-appraisal throughout the different stages of ill health. Furthermore, there are studies with contradictory findings on this subject. For example, according to Mansell et al. [125], pharmacotherapy is described as a way for “blurring the water”, since individuals under medication are not able to differentiate between their true self and their self under medication, hence unable to attribute their behavior to their personal characteristics or to antipsychotic medication [125]. In addition, in the study of Spaniol et al. [57], pharmacotherapy was interpreted as a means of remaining enslaved in mental health system. In conclusion we suggest that all these issues need to be taken under consideration when interventions are targeted on the empowerment of people living with SMI.

Moreover, despite the fact that in the present study, acceptance of pharmacotherapy was an important element in participants’ recovery, there is no data, even anecdotal, to support the issue that medication management approach by mental health professionals in Cyprus is in line with the recovery model. In particular, in Cyprus, as well as in Greece [128] there still exists the physician centered culture, hence the consumers of mental health services are not actively involved in decision-making processes regarding their medication. As a result, medication is usually prescribed with little or no consultation and often, without consideration of the individual's wishes and experience, affecting one's willingness to accept medication or even to comply with instructions given by a mental health prescriber. Based on that, the present study highlights the importance of the implementation of the recovery model and patients’ participation in decision making, not only in Cyprus, but in all those countries which still hold a physician centered approach and power relations between mental health professionals and patients.

The data reported herein with regard to the way participants experience their living with the illness and subsequent therapy, call for the reorganization of the culture of mental healthcare policy in Cyprus towards a more recovery-oriented approach in both community and hospital organizations. Empowerment of the resilience of people living with SMI, participation in decision-making and planning for their own care, along with effective management of pharmacotherapy, need to be the ultimate focus of mental health services [119, 123]. Unfortunately, within the mental healthcare system of Cyprus there still exists disease – centered approaches, characterized by limited involvement of mental health services consumers in the design of the therapeutic plan [61].

According to international literature, transformation of mental health services towards a recovery-oriented system of care requires the collaborative work of all organizations involved in mental healthcare in order to articulate a framework and a mandatory strategy for recovery and well-being in relation to mental health problems. The proposed policy needs to address future healthcare plans, hospital accreditation standards and annual objectives for hospital and community mental health services [123]. The objectives should include: a) empowerment of individuals living with mental illness and their families, in order to be able to participate in designing their own care, as well as meet their everyday life needs in a culturally competent manner, b) promoting self-determination in people living with SMI, and c) educating healthcare professionals about the principals of recovery-oriented healthcare systems [35, 114, 117, 123].

According to Park et al. [123] such an effort may include a) quantitative and qualitative research to assess the knowledge and relevant attitudes regarding recovery and recovery-oriented practices of both healthcare professionals and consumers of mental health services, b) collaboration between consumers of mental health services and the heads of mental health professionals, as well as policy makers to develop Recovery-in-Action Initiatives to meet the needs and resources of all partners in the project and c) a systematic theory-based assessment of transformation of attitudes and practices among all groups of partners in order to identify relevant barriers and supports within the local context [123].

Another negative influence on ones’ self-appraisal necessitating the transformation of the culture of organization and provision of care in mental health services was the participants’ tragic experience upon their hospitalization in the State psychiatric hospital of Cyprus (Athalassa hospital) where involuntary hospitalization was applied during the acute phase of mental diseases. In particular, the descriptions of the participants highlighted experiences of human rights violation, even in relation to basic hygiene. Moreover, the main sources of dissatisfaction were the quality of the facilities and relationships with healthcare professionals. These findings partially support the data reported by Johansson & Lundman [129], which underscore the experience of being subjected to involuntary care as a case of restricted autonomy, violation of physical integrity and devaluation of humanity. Interestingly, it has been reported that hospitalization in acute care settings was associated with a negative impact on one’s perception on self- identity due to self- stigma [67, 69, 72, 103]. These findings support the need for interventions to improve conditions prevailing in the Athalassa hospital, as well as reform the way mental health services are provided in both hospital and community setting, in order for the consumers of mental health services to preserve a positive personal identity [113, 123, 130].

### Proposed interventions

The importance of the proposed interventions is supported by evidence which shows that negative self-appraisal in people living with mental illness may be related to poor hope regarding recovery and social interaction and subsequent self stigma. In particular, such data illustrate that people exhibiting high degree of self-stigma and at the same time a high level of insight report poor hope regarding recovery, low self-esteem and limited social interactions compared to people with high insight and low levels of self-stigma [103]. Based on these findings, interventions targeted at social stigma arising from mental illness, and empowerment of mentally ill patients’ self-esteem seem relevant, along with interventions aiming to alleviate self-stigma. Additionally, the participants in the present study emphasized the therapeutic influence of the community mental health services on their existence in terms of socialization, creativity and rehabilitation and subsequently on the development of a positive self-view. Similar findings were presented in the study by Sun Kyung & Eun Hee [109], where attending a clubhouse enhanced patients’ sense of belonging and their recovery.

International literature supports the implementation of specific types of psychotherapy addressing self-identity issues in order to assist people living with SMI towards recovery. For example, the study by Bargenquast & Shweitzer [108] provides evidence for the effectiveness of Meta-cognitive Narrative Psychotherapy regarding self-appraisal issues during the process of recovery. Meta-cognitive Psychotherapy is an innovative type of psychotherapy developed by Lysaker et al. [110], where meta-cognition is a means to understand how one goes from discrete perception into an integrated representation of self and others [131]. As for people living with SMI, it is proposed that this type of psychotherapy may empower the ability for self-reflection, decrease hallucinations and delusions and improve insight [103, 131–133]. Moreover, therapeutic recreation is a proposed model of therapy aiming to enhance the level of self-determination in individuals living with SMI [114]. Self-determination refers to the motivation of an individual influenced by three psychological needs: a) competence (feeling of success or optimal challenge), b) autonomy (provided choice and control over a particular behavior) and c) relatedness (feeling socially connected to others). Overall, self-determination requires the satisfaction of all the aforementioned needs in order to be developed. Medication adherence is strongly related to self-determination [114].

Another type of therapy proposed is the model of Acceptance and Commitment Therapy (ACT). The aim of this therapy is to achieve a balance between acceptance of what is thought to be impossible to change and commitment to actions, a process which could support individuals living with SMI in transforming their goals and finding meaning beyond the consequences of sustained symptoms and impairments [122]. Moreover, mental healthcare professionals are expected to support individuals living with SMI in overcoming obstacles and deciding on their values.

In terms of enhancement of adherence to therapy, both pharmacological and psychosocial, literature provides substantial evidence for the effectiveness of psychosocial interventions [133] by educating patients and their families on both disease pathophysiology and the effective management of the adverse side effects of medication [134, 135]. Such studies address people with mental illness, as well. In addition, there is empirical evidence that supports the implementation of Cognitive Behavioral Therapy psychotherapeutic interventions to promote adherence to pharmacotherapy and psychosocial functioning [136, 137]. The main issues that are usually addressed include: a) a broad discussion about the reasons behind patient’s acceptance or refusal to take medication and the advantages and disadvantages following his/her decision, b) reattribution of patients’ thoughts to hallucinations believed to enhance acceptance and adherence to medication, and c) the involvement of patient in decision-making [138].

### Facilitators of recovery

A well described facilitator of recovery by the participants regarded the effective and therapeutic relationship between patients and mental health nurses, the importance of which is underlined in many studies. The significance of empathic and supportive relationships between consumers of mental health services and health professionals lies on evidence which shows that these qualities enhance understanding of healthcare professionals and reassurance towards people living with SMI. This in turn seems to make patients feel accepted, facilitating their social integration [54, 124, 139–143]. Overall, empathy, respect and availability provided by health professionals have been described in international literature as basic elements of the therapeutic relationship [47, 67, 104]. On the contrary, unavailable and depersonalized mental health professionals have been described as a barrier to recovery [143]. More importantly, the participants in the present study described the particular phases of the relationship between them and mental health nurses as a dynamic process, while mutual acceptance and empathic understanding were crucial in maintaining a positive self-image by both sides.

Additionally, education of healthcare professionals in order to apply recovery-oriented models of therapy is needed. There are particular objectives mentioned in the literature that need to be supported through the implementation of the recovery model by mental healthcare professionals, especially nurses, such as: a) navigation of people living with mental illness on their journey to recovery, b) believing in the support and recovery of people living with mental illness [47, 144], and c) provision of information, choice, practical support on financial matters and employment of people living with SMI.

However, international literature [35, 145], underlines a lack in the training of mental health nurses when it comes to applying the recovery model, although they seem to have the knowledge to implement it [35, 145]. Furthermore, while mental health nurses show confidence in their understanding of the importance of the recovery model [145], they are uncertain about providing practical support for employment and helping mentally ill patients find a house or manage their own symptoms [35, 145], areas where more education is necessary.

Another important finding, although beyond the scope of the present study, was the therapeutic effect of the research interview for the participants. It seemed that participants considered the interview to be ‘redemptive’, since they used phrases such as “I expelled some residues off me, which were useless’. The therapeutic effect of the phenomenological research interview has been considered in previews research [146]. In particular, studies underline the importance of the research interview for the development of trust and rapport between the researcher and the participants in order for the latter to be able to disclose their pure experience with the phenomenon under study. This dynamic seemed to endorse the participants of the present study to reflect on the way mental illness and its therapy made them feel, a procedure which proved to be therapeutic [147]. Nevertheless, the ultimate goal of phenomenological research is for the participants to be encouraged to relive their personal experiences in order to acknowledge their inner meanings and reflections [147].

## Conclusions

The present study provides an insight into the way people living with SMI interpret their self and adhere to pharmacotherapy in Cyprus. These data may be useful for the formation of psychotherapeutic interventions aiming to alleviate suffering following mental disease, enhance positive self values, as well as improve adherence to therapy, and at the same time to empower patients’ self -image through the different phases of the illness within the special context of Cyprus mental health services. Moreover, the present findings provide a deeper understanding of relevant issues addressed previously in the literature. The present data also illuminate the importance of empathic understanding for healthcare professionals with regard to concerns and particular needs of individuals living with SMI. Above all, our findings signify the necessity to transform the current mental healthcare services provided in Cyprus into a more recovery- oriented approach, embracing personal identity issues and effective management of pharmacotherapy. Moreover, future qualitative studies are also proposed to further explore self-identity issues during ill health, as well as their association with adherence to therapy.

### Limitations

Although demographic and clinical characteristics of the participants were more or less similar to those of the majority of the users of the community mental health service where data collection took place, it should be noted that approximately half of the users of the service could not be reached, mostly because they were in relapse and hence unable to visit the service or enter the study even if they wished so. Another limitation might be that the participants were recruited from the same service, thus one may argue that data collected herein represent solely the consumers of the community mental health service of the particular province. However, even though the participants at the time of data collection were using the particular community service, they all had a prior experience of inpatient and outpatient mental health services all over Cyprus due to the severity and long term course of their illness. Most importantly, the researchers of the current study, wish to emphasize that while the participants were fully informed about the scope and the research questions under study, nevertheless they all had already accepted the diagnosis of severe mental illness for themselves. As a result, the present findings may not be generalized to any population of individuals living with SMI who do not accept the label of such a diagnosis for themselves for any reason, including perception of personal empowerment, lack of insight, etc.

## Abbreviations

ICD-10: International Statistical Classification of Diseases and Related Health Problems. 10th Revision
DSM-IV-TR: Diagnostic and statistical manual of mental disorders, Fourth Edition (Text Revision)
DSM-5: Diagnostic and statistical manual of mental disorders, 5th Edition
PHMNs: Psychiatric health mental nurses
SMI: Severe mental illness
